# Spatiotemporal Variations in Seed Set and Pollen Limitation in Populations of the Rare Generalist Species *Polemonium caeruleum* in Poland

**DOI:** 10.3389/fpls.2021.755830

**Published:** 2022-01-03

**Authors:** Justyna Ryniewicz, Katarzyna Roguz, Paweł Mirski, Emilia Brzosko, Mateusz Skłodowski, Ada Wróblewska, Beata Ostrowiecka, Izabela Tałałaj, Edyta Jermakowicz, Marcin Zych

**Affiliations:** ^1^Botanic Garden, Faculty of Biology, University of Warsaw, Warsaw, Poland; ^2^Faculty of Biology, University of Białystok, Białystok, Poland

**Keywords:** *Apis mellifera*, plant-pollinator interactions, pollen limitation, pollinator composition, reproductive ecology, seed set, spatiotemporal variation

## Abstract

A vast majority of angiosperms are pollinated by animals, and a decline in the number and diversity of insects often affects plant reproduction through pollen limitation. This phenomenon may be particularly severe in rare plant species, whose populations are shrinking. Here, we examined the variability in factors shaping reproductive success and pollen limitation in red-listed *Polemonium caeruleum* L. During a 5-year study in several populations of *P. caeruleum* (7–15, depending on year), we assessed the degree of pollen limitation based on differences in seed set between open-pollinated (control) and hand-pollinated flowers. We analysed the effects of flower visitors, population size, and meteorological data on plant reproductive success and pollen limitation. Our study showed that pollen limitation rarely affected *P. caeruleum* populations, and was present mainly in small populations. Pollen limitation index was negatively affected by the size of population, visitation frequency of all insects, and when considering the visitation frequency of individual groups, also by honeybee visits. Seed production in control treatment was positively influenced by the population size, average monthly precipitation in June and visits of hoverflies, while visits of honeybees, average monthly temperature in September, and average monthly precipitation in August influenced seed production negatively. As generalist plant *P. caeruleum* can be pollinated by diverse insect groups, however, in small populations their main visitors, the honeybees and bumblebees, may be less attracted, eventually leading to the disappearance of these populations. In pollination of *P. caeruleum* managed honeybees may play a dual role: while they are the most frequent and efficient flower visitors, their presence decreases seed set in open-pollinated flowers, which is most probably related to efficient pollen collection by these insects.

## Introduction

Mutualistic interactions between plants and their pollinators play pivotal roles in the functioning of terrestrial ecosystems and global food production. Almost 90% of angiosperms are pollinated by animals (Ollerton et al., 2011), mainly insects, and their presence is essential for effective pollination. Recent decline in the number and diversity of insects has been particularly severe (Hallmann et al., 2017; Lister and Garcia, 2018; Wagner et al., 2021; Warren et al., 2021), and it has negatively affected many other groups of organisms, thus destabilising ecosystem functioning (Thomas et al., 2019; van der Sluijs, 2020). Another causes of disturbances in ecosystem functioning and interactions among its components are habitat loss and climate change. Habitat loss results in the disappearance of plant populations and their isolation (i.e habitat fragmentation), thus limiting gene flow and reducing genetic variability. In the face of global warming, the timing of flowering and insect activity may change, leading to a misfit between them (Kudo and Ida, 2013; Forrest, 2015; Ziemiański and Zych, 2016). High temperature and water deficit may lead to plant wilting and low fertility, such as through seed, fruit, or anther abortion and effects on pollinator interactions (Young et al., 2004; Liu et al., 2012; Gallagher and Campbell, 2017; Borghi et al., 2019).

Insect-pollinated plants that reproduce exclusively by means of seeds may suffer from a decline in insect populations. As a consequence of a decline in insect diversity and abundance, plants may be subjected to pollen limitation, during which pollen grains of the lowest quantity and quality (i.e self or otherwise incompatible pollen) are delivered on the stigmas by insects, resulting in the production of fewer fruits or seeds than normally produced with adequate pollen receipt (Ashman et al., 2004; Knight et al., 2005).

Pollen limitation is among the main causes of decrease in seed and fruit production, which further affects the stability of plant populations (Jennersten and Nilsson, 1993; Ashman et al., 2004; Aizen and Harder, 2007). This phenomenon affects many plant species, and it may arise as a result of several factors, such as plant life history, phylogenetic history, mating system, and ecological aspects (Ashman et al., 2004). The major consequences of inadequate pollen receipt on the stigma include the selection acting on mating system and floral traits, in addition to changes in the abundance of individuals in the population (Knight et al., 2005). In particular, small populations may be affected by pollen limitation due to the presence of fewer potential mates and/or pollinators (Ågren, 1996; Knight et al., 2005; Zhang and Lou, 2015). Although most plant species are generalists, the majority of the studies on pollen limitation were focused on specialist plants (Waser et al., 1996).

To investigate the causes and consequences of pollen limitation in a generalist plant, we selected *Polemonium caeruleum* – a rare, red-listed species, whose some of populations are shrinking in its Polish range. In Poland, fragmentation of *P. caeruleum* populations, loss of pollinators, and presence of mixed mating systems (both self-compatible and self-incompatible populations) indicate that this species is adapting to the changing environment. According to a previous study on a single population of *P. caeruleum*, pollen limitation may affect even larger populations of this species (Zych et al., 2013); however, studies involving other populations did not confirm this phenomenon (Ostrowiecka et al., 2017). In addition, these populations differ in terms of insect assemblages visiting flowers (Zych et al., 2013; Ostrowiecka et al., 2017) and chemical composition of nectar (Ryniewicz et al., 2020).

Previous reports regarding the pollination and reproductive biology of *P. caeruleum* encouraged us to continue and expand this research by focussing on factors that shape seed production and pollen limitation among populations in the Polish range of the species. Pollinator service (pollinator assemblage composition and visitation frequency) is one of the most immediate factors affecting pollen limitation (Motten, 1986; Gómez et al., 2007, 2010), and it may differ significantly in time and space (Gómez et al., 2010; Fernández et al., 2012; Zych et al., 2019). Therefore, we tested (i) whether insect assemblages visiting flowers are relatively stable or variable in a given population and (ii) how insect assemblages and changes in their composition affect seed set and pollen limitation. Small fluctuations in the communities of insects visiting flowers may prove the adaptation of plants to a specific group of pollinators. We also explored the spatiotemporal variations in pollen limitation to establish its probable long-term effects, trends, and threats among the studied populations. In particular, small populations may be at a high risk, as individuals may be more prone to the effects of stochastic environmental processes and inbreeding depression. In some cases, decline in the number of individuals in a population may drastically reduce reproductive potential, disproportionate to the decline in the population size (the so-called Allee effect; Stephens and Sutherland, 1999).

## Materials and Methods

### Study Species

*P. caeruleum* L. (Jacob’s ladder; Polemoniaceae) is a perennial plant distributed in the temperate zone of the Northern Hemisphere. Poland represents the southernmost point of its distribution range, with most populations distributed in the northeast of the country (Rutkowski, 2000). This region is characterised by continental climate with lower mean annual temperatures compared to other regions of the country.

*P. caeruleum* produces a corymbose inflorescence with a few to over a dozen simultaneously opening, fragrant flowers with radial symmetry. The blue corolla is composed of five petals, surrounding five stamens with orange anthers and the pistil terminating in a three-lobed stigma. Most of the flowers are hermaphrodite and protandrous; however, overlapping sexual phases are common (Zych et al., 2013). The male phase is shorter than the female phase (1.7 ± 0.9 versus 2.0 ± 0.8 days; Zych et al., 2013). Previous studies on three *P. caeruleum* populations, which were also included in the present study, revealed that the Polish populations of this species are characterised by different reproductive systems, two of which are self-incompatible and one is self-compatible (Zych et al., 2013; Ostrowiecka et al., 2017). According to Pigott (Pigott, 1958), individual plants live for at least 10 years and reproduce by seeds; vegetative reproduction does not occur.

Insects visiting *P. caeruleum* flowers are attracted to pollen, nectar, and sweet scent. The study species is characterised by a generalist pollination system – flowers are usually visited by a broad range of insects, mainly social bees, flies, beetles, and butterflies (Zych et al., 2013; Ostrowiecka et al., 2017). *P. caeruleum* is a pollinator-dependent species, as pollinators are the key factor determining reproductive success (Ostrowiecka et al., 2017); consequently, lack of pollinators may lead to pollen limitation.

Land drainage, climate change, and low abundance and diversity of pollinators negatively affect *P. caeruleum* populations. Consequently, the number of plants tends to decrease, which can lead to the complete disappearance of populations (Rutkowski, 2000; Zych et al., 2013). Additionally, high temperatures and periodic droughts, which are particularly detrimental to seedling survival (Pigott, 1958), have become common in Poland in recent years (Kundzewicz and Matczak, 2012). As a result of a significant decline in populations, *P. caeruleum* is included in the Polish Red List of Plants (VU category; Kaźmierczakowa et al., 2016).

### Study Populations

**Figure 1:**
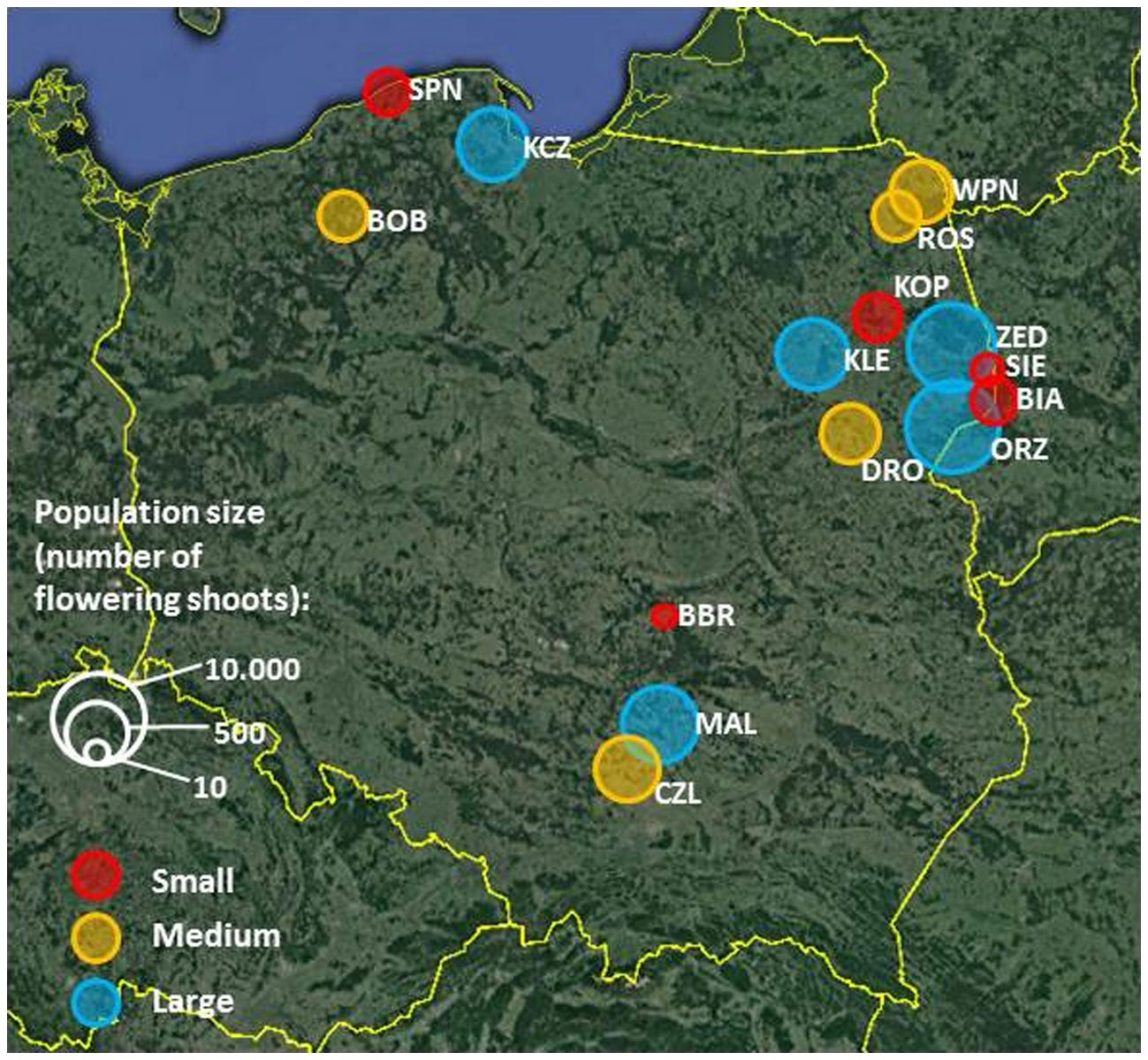
Distribution of *Polemonium caeruleum* study populations. The size of the circles and colours correspond to the average (over the study period) log-transformed size of populations (number of flowering shoots). Small populations (10–100 flowering shoots per population) are marked with red circles, medium populations (120–525 flowering shoots per population) with yellow, and large populations (775–10,000 flowering shoots per population) with blue.

The experiments involved 15 *P. caeruleum* populations of various sizes (both in terms of the number of individuals and area occupied; Figure 1) and comprising different types of plant communities distributed across the country. In the present study, we included all Polish populations of *P. caeruleum*, known from the literature, which were within the walking distance (<2 km) from roads accessible by a 4WD car. The population size was defined as the number of flowering shoots estimated every year at the peak of the flowering season (10 to 15,000 individuals per population; Supplementary Table S1).

Populations occupied different habitats: majority occurred in floristically rich wet meadows (BBR, BIA, BOB, CZL, DRO, KCZ, KLE, MAL, ORZ, WPN, and ZED), two occurred in forests or in their immediate vicinity (KOP and SIE), one occurred in a fen (ROS), and one occurred on a peninsula surrounded by sedgelands and a lake (SPN; Supplementary Table S1). Of these, three populations were located in national parks, where human activities are restricted (KOP, SPN, and WPN).

### Pollen Limitation Experiment

To assess the extent to which pollen limits seed production in *P. caeruleum* and spatiotemporal variations in this phenomenon, if any, we performed supplementary pollination experiments in all populations. During 2014–2018, at the peak of flowering (June to July in Poland), we randomly selected and marked approximately 40–50 flowers with receptive (open) stigmas from different individuals in each population. There were fewer experimental flowers in smaller populations (<100 flowering shoots). Half of the marked flowers were left for open pollination, and the rest were hand-pollinated with pollen obtained from another individual growing at a minimum distance of 3 m. Hand pollination was performed by brushing the dehisced anthers against the stigma using tweezers. The procedure was continued until the stigma was covered with pollen.

At 4–5 weeks after pollination, mature fruits were collected, and seeds were counted under a stereomicroscope. Empty seeds (aborted seeds) were excluded from the analysis. Depending on the year and population, we noted the different degree of fruit damage caused by herbivores. In some populations, we could collect almost all experimental fruits, while in others, only 30% of the fruits were undamaged. Damaged fruits and seeds were excluded from the analysis.

The pollen limitation (PL) index – a measure of the magnitude of pollen limitation – was calculated using the following formula:


PL=(Ps−Po)/Pmax

where Ps is the number of seeds from the hand-pollination treatment, Po is the number of seeds from the control treatment, and Pmax is the larger of the two values (Baskin and Baskin, 2018).

The PL index was calculated for each population in a given year; thus, the values are always expressed at the population level. The PL index should range from zero, when both treatments produce the same number of seeds, to one, when natural pollination (control) does not produce seeds (complete pollen limitation). In the present study, however, in 23 of the 56 cases, the PL index was below zero, and we decided to include the negative values in further analysis, as we assumed that raising only the negative values to zero will reduce the reliability of the data.

### Insect Visitors

To assess the assemblage composition of insects that visited *P. caeruleum* populations, we performed observations of insect activity on flowers. During 2015–2018, we recorded insect activity simultaneously with the experiments set up to assess pollen limitation. To determine the frequency of insect visitation to flowers, we applied the method described by Zych et al. (2013). We randomly selected a patch of flowering plants, with two to five shoots in full bloom, and recorded insects visiting flowers for 15 min using a digital camera (HC-VX870; Panasonic Corp.) set on a tripod at ~1.0–1.5 m from the plants; if possible, we attempted to record a different patch each time. In each year, we obtained 12 recordings of insect activity for at least 2 days (180 min^−1^·population^−1^·year^−1^) in each population. Observations were recorded from 10.00 to 16.00 h (peak pollinator activity) under appropriate weather conditions; we avoided recording on rainy and windy days. In the laboratory, each insect recorded visiting a flower was counted and classified into the following groups: (i) honeybees (*Apis mellifera*), (ii) bumblebees (*Bombus* spp.), (iii) solitary bees, (iv) hoverflies (Syrphidae), (v) other flies (other Diptera), (vi) butterflies (Lepidoptera), (vii) beetles (Coleoptera), and (viii) others (e.g., wasps and members of orders Neuroptera, Hemiptera, and Orthoptera). We only considered insects that touched the flower sex organs as potential pollinators. The frequency of insect visitation was calculated per census (15 min) relative to the number of inflorescences in a recorded patch.

### Meteorological Data

To determine the effects of meteorological conditions on seed production and the PL index, we collected meteorological data, including mean monthly precipitation and ambient temperature, from the nearest weather station (Institute of Meteorology and Water Management).^1^ We included meteorological data from May to July of the same year in which the seed experiment was conducted. Additionally, we included meteorological data from August to October of the previous year to determine the effect of weather on the condition of plants and accumulation of resources. For instance, favourable conditions for the growth of perennials in autumn may delay the initiation of cold hardiness, which triggers the storage of assimilates in roots (McKenzie and McLean, 1980).

### Statistical Analysis

We used R 4.0.3 for data analyses. First, data were assessed for normality (Shapiro–Wilk test) and transformed when necessary and possible. To determine the differences in seed production and frequency of insect visitation to flowers across populations and years, we applied two-way ordinal ANOVA using the “clm” function in the “ordinal” package, followed by the “Anova” function in the “car” package, because the assumption of data normality for parametric tests was not met, even when the data were appropriately transformed.

To determine the significance of differences in seed set between hand-pollinated and naturally pollinated flowers among populations in a given year (i.e., populations experiencing pollen limitation), we applied a nonparametric Mann–Whitney U-test.

Prior to running the models, we checked for multicollinearity among the variables. As the assumption of data normality was not met for most variables, we performed Spearman’s pairwise correlation analysis, and excluded variables that were strongly correlated (*r* ≥ |0.7|) (Hosmer and Lemeshow, 2000) followed by variance inflation factor (VIF) stepwise selection, and excluded variables with VIF ≥ 5 (Zuur et al., 2010). Finally, variables were centred and scaled to adopt a similar range of values to improve model performance.

Generalised linear mixed model (GLMM) was used to evaluate the effects of the frequency of visitation by specific insect groups and meteorological variables on seed production in control treatment. In the model log-transformed population size, the mean frequency of visits by specific insect groups, and temperature and precipitation in selected months were included as the fixed effects and population was included as the random effect. The effect of year was not included in the model, due to near-zero variance and negligible role of the random effect of the year in relation to the random effect of the population.

During the selection of the variables to GLMM, characterizing meteorological conditions, we avoided rejecting variables describing precipitation and temperature during months in which the seed experiments were set, as we found them more influential. Finally, all variables were centred and scaled to adopt a similar range of values to improve model performance. As the data on seed set included many zero values and were characterised by negative binomial distribution, we applied zero-inflated negative binomial GLMM using the “glmmTMB” function in the “glmmTMB” package (Brooks et al., 2017), as it provided a better fit than the other models [as evidenced by a lower Akaike information criterion (AIC) value] and prevented overdispersion. We selected the best models based on AIC. The models developed during the analyses are presented in Supplementary Data (Supplementary Table S2).

Furthermore, we applied a linear mixed model using the “lmer” function in the “lme4” package to analyse the possible predictors affecting the PL index, as it was characterised by normal distribution. In two models, the calculated values of the PL index for each population in each year were combined with the (i) average frequency of insect groups visiting flowers and (ii) average monthly meteorological variables for each month, including population and year as the random effects. As zero models in both cases were characterised by the lowest AIC value, we averaged the models. For this purpose, we compared the AICc values of the candidate models, and averaged those including factors with ΔAICc <2 (compared with the zero model, characterised by the lowest AICc). Finally, only one factor was included in each model: frequency of visits by honeybees and temperature in October (ΔAICc values of the candidate models are presented in Supplementary Tables S3 and S4).

To determine whether the PL index is shaped by the population size and the frequency of insect visits, we performed linear regression analysis using the “lm” function in the “stats” package. We also performed linear regression analysis to determine whether the frequency of insect visitation was affected by population size, after transforming the dependent variable (frequency of insect visitation) to a normal distribution by adding 0.5% to each score, followed by log-transformation.

## Results

### Pollen Limitation

The number of seeds produced differed among populations and years in both control (χ^2^ = 803.70, df = 14, *p* < 0.001 and χ^2^ = 798.79, df = 4, *p* < 0.001, respectively) and hand-pollinated flowers (χ^2^ = 809.26, df = 14, *p* < 0.001 and χ^2^ = 758.62, df = 4, *p* < 0.001, respectively).

**Figure 2:**
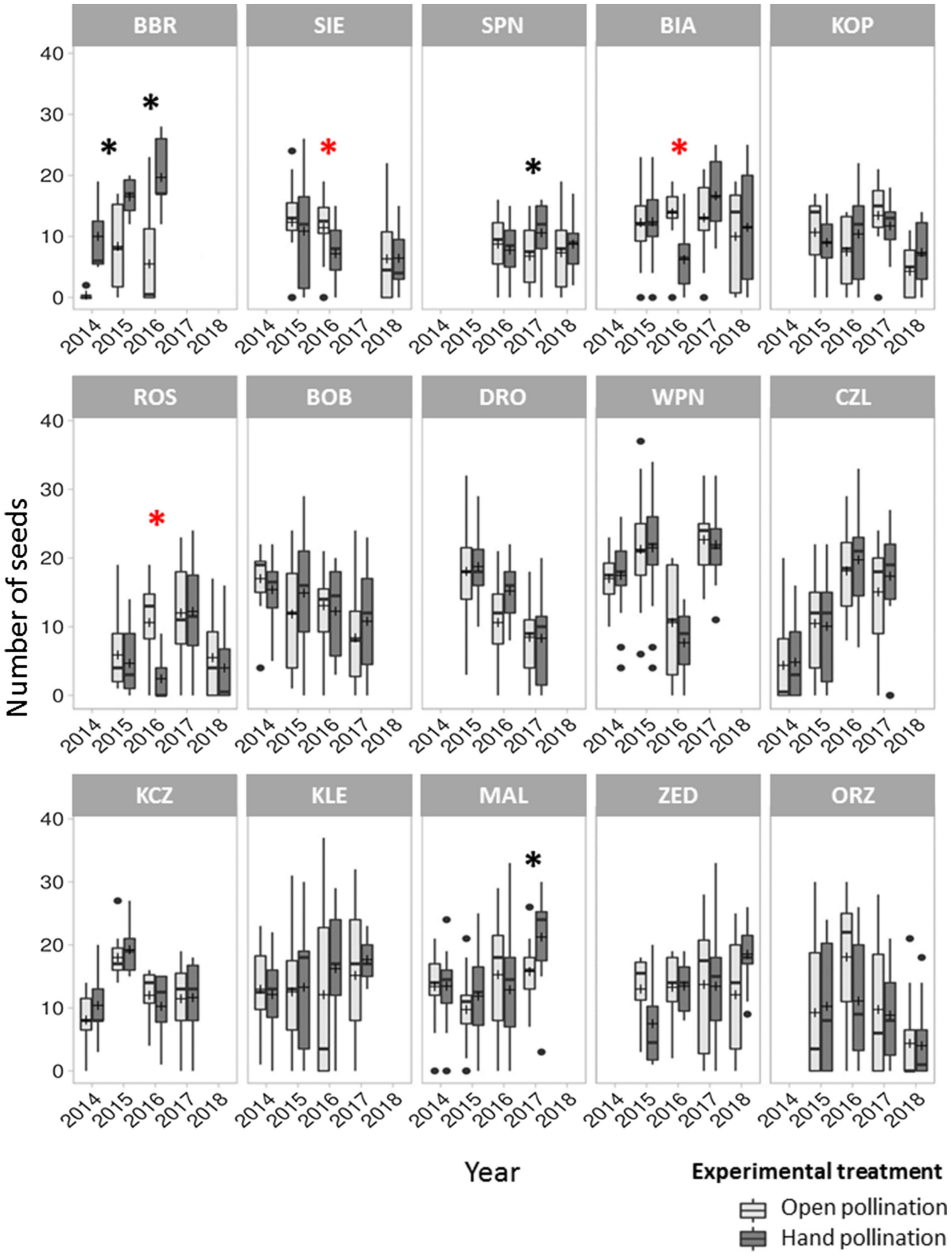
Mean seed number per fruit under the open pollination (control) and hand-pollination treatments in 15 *Polemonium caeruleum* populations over 5 years. Graphs on the **top**, in the **middle**, and at the **bottom** represent the small, medium, and large populations, respectively. Error bars indicate SE; lines inside the bars indicate median; and crosses inside the bars indicate mean. Significant differences in number of seeds between the control and hand-pollinated treatments (*p* < 0.05) are marked with black asterisks (*). Cases where the number of seeds was significantly higher in the control treatment than in the hand-pollinated treatment are marked with red asterisks (*).

The mean number of seeds per fruit was, respectively, 11.9 ± 8.0 and 12.7 ± 7.9 in the control and hand-pollinated treatments, indicating 6.3% increase with the latter, although this difference was not significant. However, seed production significantly differed among populations and years, indicating that pollen limitation was temporally present in at least three populations (Figure 2). Pollen limitation primarily affected small populations; however, this phenomenon was also observed in one large population (MAL). Additionally, in the three affected populations, control flowers produced significantly more seeds than hand-pollinated flowers (Figure 2). The degree of pollen limitation varied across populations and years, ranging from −0.77 to 0.96, with mean of 0.04. Moreover, 23 of the 56 PL index values in *P. caeruleum* populations were below zero.

### Insect Visitors

**Figure 3:**
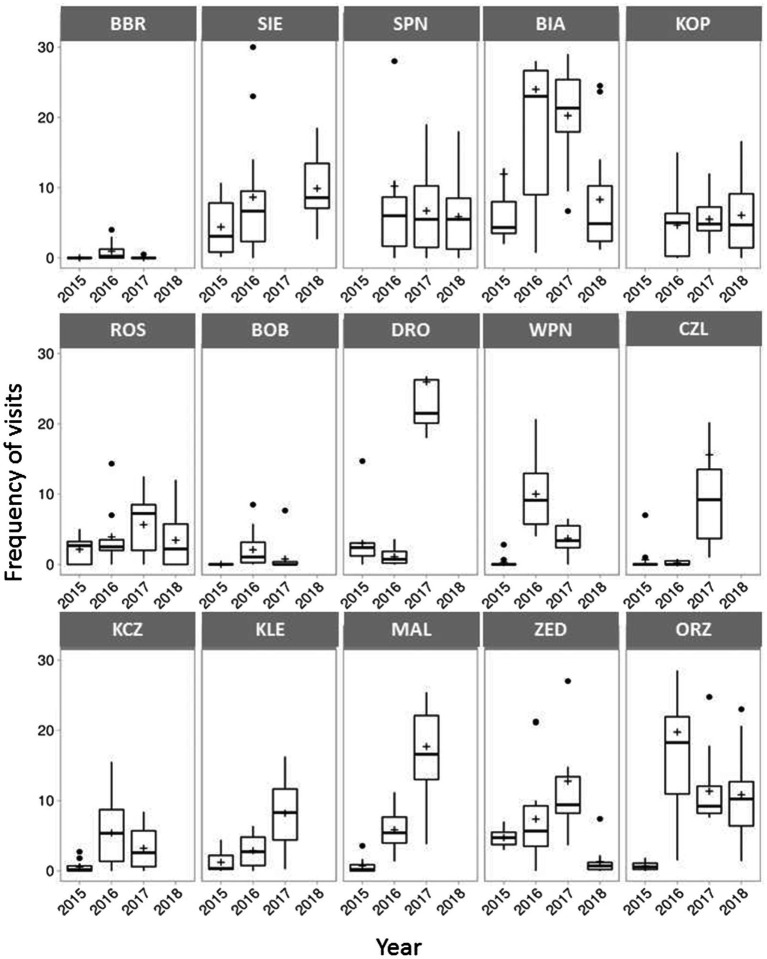
Mean frequency of total insect visits in 15 *Polemonium caeruleum* populations over 4 years. Graphs on the **top**, in the **middle**, and at the **bottom** represent the small, medium, and large populations, respectively. Error bars indicate SE; lines inside the bars indicate median; and crosses inside the bars indicate mean.

The overall insect visitation frequency was 6.9 ± 8.9 visits per census (15 min) per inflorescence. The frequency of insect visits significantly differed among populations (χ^2^ = 151.676, df = 14, p < 0.001) and years (χ^2^ = 52.133, df = 3, *p* < 0.001; Figure 3).

**Figure 4:**
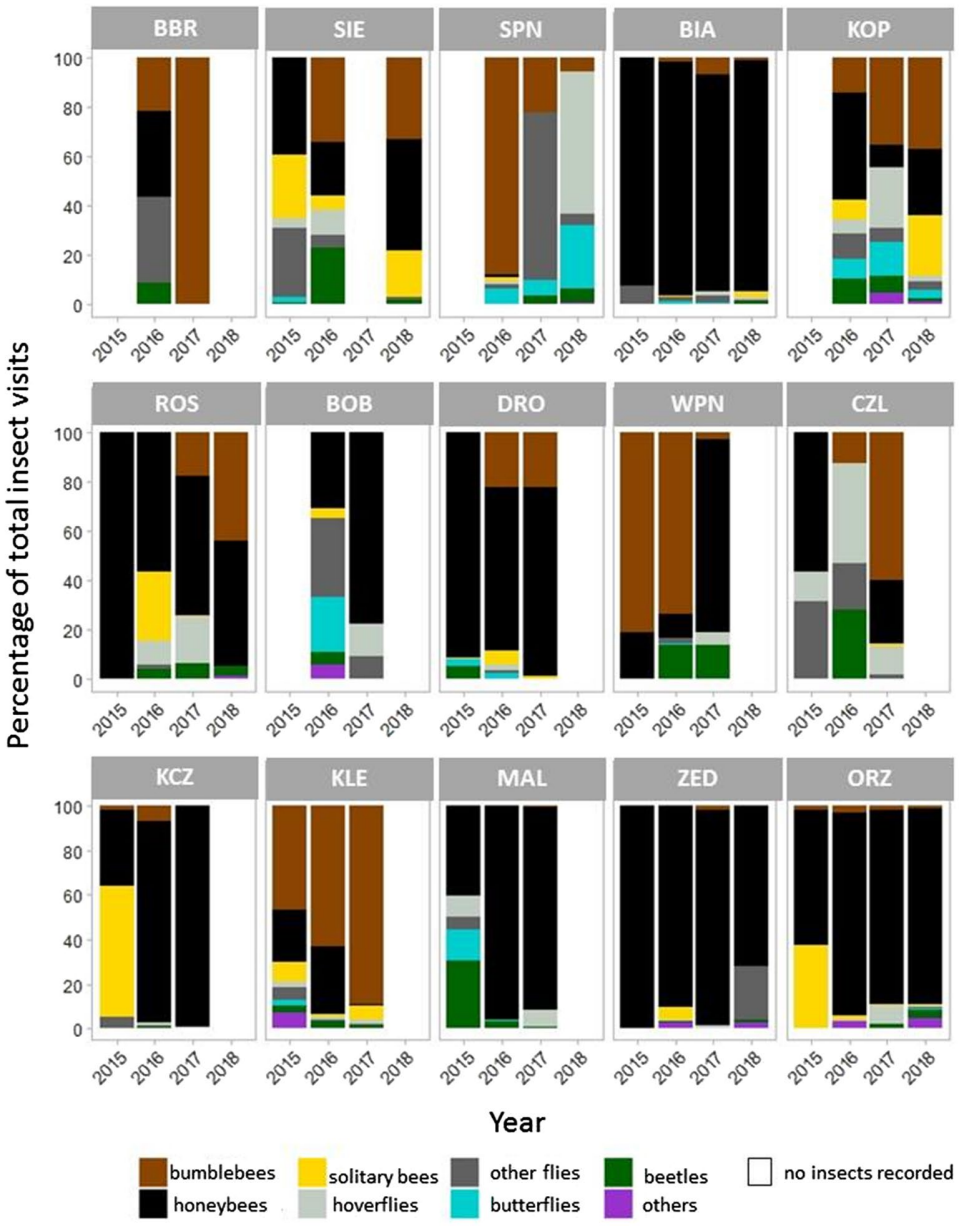
Taxonomic diversity of insects visiting *Polemonium caeruleum* flowers in 15 populations. Graphs on the **top**, in the **middle**, and at the **bottom** represent the small, medium, and large populations, respectively.

During 143 h of recording, we observed 13,553 insect visits on *P. caeruleum* flowers, and honeybees and bumblebees accounted for over 70 and 17% of all visits, respectively. Dipterans, lepidopterans, and coleopterans accounted for only 5, 1.2, and 2% of all visits, respectively (Figure 4).

Honeybees were the most frequent flower visitors among the recorded insects, and some populations were visited almost exclusively by honeybees (Figure 4). Meanwhile, in some populations, bumblebees were the predominant visitors in some years. The populations located in national parks (KOP, SPN, and WPN) were visited by more diverse insect groups.

### Meteorological Observations and Population Size

The highest mean monthly temperatures were recorded in populations in the south of the country (BBR: 15.2°C; MAL: 15.0°C; and CZL: 14.9°C) and in the population in the northeast (DRO: 15.0°C). In contrast, the lowest temperatures were recorded in populations in the north of the country (WPN: 13.9°C; KCZ: 14.0°C; and ZED: 14.2°C).

Regarding precipitation, less pronounced trends were noted. The highest precipitation was recorded in two population in the north (SPN: 63.5 mm and BOB: 61.8 mm) and one population in the northeast (ZED: 61.0 mm), and the lowest precipitation was recorded in populations in the northeast (SIE: 44.2 mm; KLE: 46.3 mm; and KOP: 49.8 mm). Across years, plants that produced seeds in 2018 were exposed to the highest temperatures, whereas plants that produced seeds in 2015 were exposed to the lowest precipitation.

The number of flowering shoots varied among populations, and while this number remained relatively stable in most of the populations, there were marked fluctuations in some populations (e.g., ZED, MAL, KCZ, DRO, and BOB) during the study period (Supplementary Table S1).

### Effects of Insect Visitation Frequency, Meteorological Conditions, and Population Size on Seed Set and Pollen Limitation

The GLMM testing the response of number of seeds in control treatment showed a positive effect of population size (GLMM, *p* = 0.014), the visitation frequency of hoverflies (GLMM, *p* = 0.02), and average precipitation in June (GLMM = 0.046). On the other hand, the visitation frequency of honeybees, precipitation in August of the previous year, and average temperature in September of the previous year (GLMM, *p* = 0.041, *p* = 0.042, *p* = 0.012, respectively) negatively affected seed production in control treatment (Table 1). The difference between the value of R2 marginal (describing the proportion of variance explained by the fixed variables), and R2 conditional (describing the proportion of variance explained by both the fixed and random variables), indicate that the share of variance explained by the random variable (populations) in the model was moderate.

**Table 1:** Results of zero-inflation generalised linear mixed models with negative binomial distribution considering population as the random effect for testing the response of seed set of *Polemonium caeruleum* in control treatment to the frequency of visit by different insect groups and meteorological conditions.

	Seed set		
Predictors	Log-mean	CI	*p*
(Intercept)	2.61	2.53 to 2.69	<0.001
Population size	0.11	0.02 to 0.20	**0.014**
Honeybee	−0.08	−0.16 to −0.00	**0.041**
Bumblebee	−0.03	−0.08 to 0.02	0.195
Solitary bees	−0.02	−0.10 to 0.06	0.646
Hoverflies	0.12	0.02 to 0.22	**0.020**
Other flies	0.01	−0.07 to 0.08	0.880
Butterflies	−0.07	−0.19 to 0.04	0.195
Beetles	−0.03	−0.10 to 0.04	0.368
Other insects	0.02	−0.05 to 0.08	0.587
Precipitation in May	0.03	−0.04 to 0.10	0.420
Precipitation in June	0.10	0.00 to 0.20	**0.046**
Precipitation in July	−0.05	−0.12 to 0.03	0.226
Temperature in July	−0.04	−0.14 to 0.06	0.409
Precipitation in August of the previous year	−0.09	−0.17 to −0.00	**0.042**
Precipitation in September of the previous year	0.03	−0.07 to 0.13	0.575
Temperature in September of the previous year	−0.10	−0.17 to −0.02	**0.012**
Precipitation in October of the previous year	−0.08	−0.16 to 0.00	0.060
Temperature in October of the previous year	−0.04	−0.15 to 0.07	0.485
Random Effects			
σ^2^	0.34		
τ_00 population_	0.02		
N _population_	15		
Observations	636		
Marginal R^2^/Conditional R^2^	0.121 / 0.161		

Significant effects are marked with boldface. σ^2^: residual variance of the random effect; τ_00_: random intercept variance.

The linear mixed models demonstrated that the PL index was negatively affected by the visitation frequency of honeybees (Table 2A) and positively affected by ambient temperature in October (Table 2B). In both models, among the random variables, population explained more variation than year; however, in general, the random variables had little effect. The value of the R^2^ marginal was much lower than that of R^2^ conditional, indicating that the random variables (years and populations) in the model explained most of the variation.

**Table 2:** Results of linear mixed models testing the response of the pollen limitation (PL) index of *Polemonium caeruleum* calculated for each population in each year of the study to the frequency of insect groups visiting flowers **(A)** and meteorological conditions in each study month **(B)**, considering population and year as the random effects.

	PL index		
Predictors	Estimates	CI	*p*
(A)			
(Intercept)	0.05	−0.06 to 0.16	0.370
honeybee	−0.10	−0.17 to −0.03	**0.007**
Random effects			
σ^2^	0.04		
τ_00 Population_	0.02		
τ_00 Year_	0.00		
N _Population_	15		
N _Year_	4		
Observations	47		
Marginal R^2^/Conditional R^2^	0.146/0.451		
(B)			
(Intercept)	0.05	−0.05 to 0.16	0.314
Temperature October previous year	0.11	0.03 to 0.19	**0.006**
Random effects			
σ^2^	0.04		
τ_00 Population_	0.02		
τ_00 Year_	0.00		
N _Year_	5		
N _Population_	15		
Observations	55		
Marginal R^2^/Conditional R^2^	0.158/0.440		

After model averaging, only one factor could be included in each model as the predictor: frequency of visits by honeybees and temperature in October. Significant effects are marked with boldface. σ^2^: residual variance of the random effects; τ_00_: random intercept variance.

**Figure 5:**
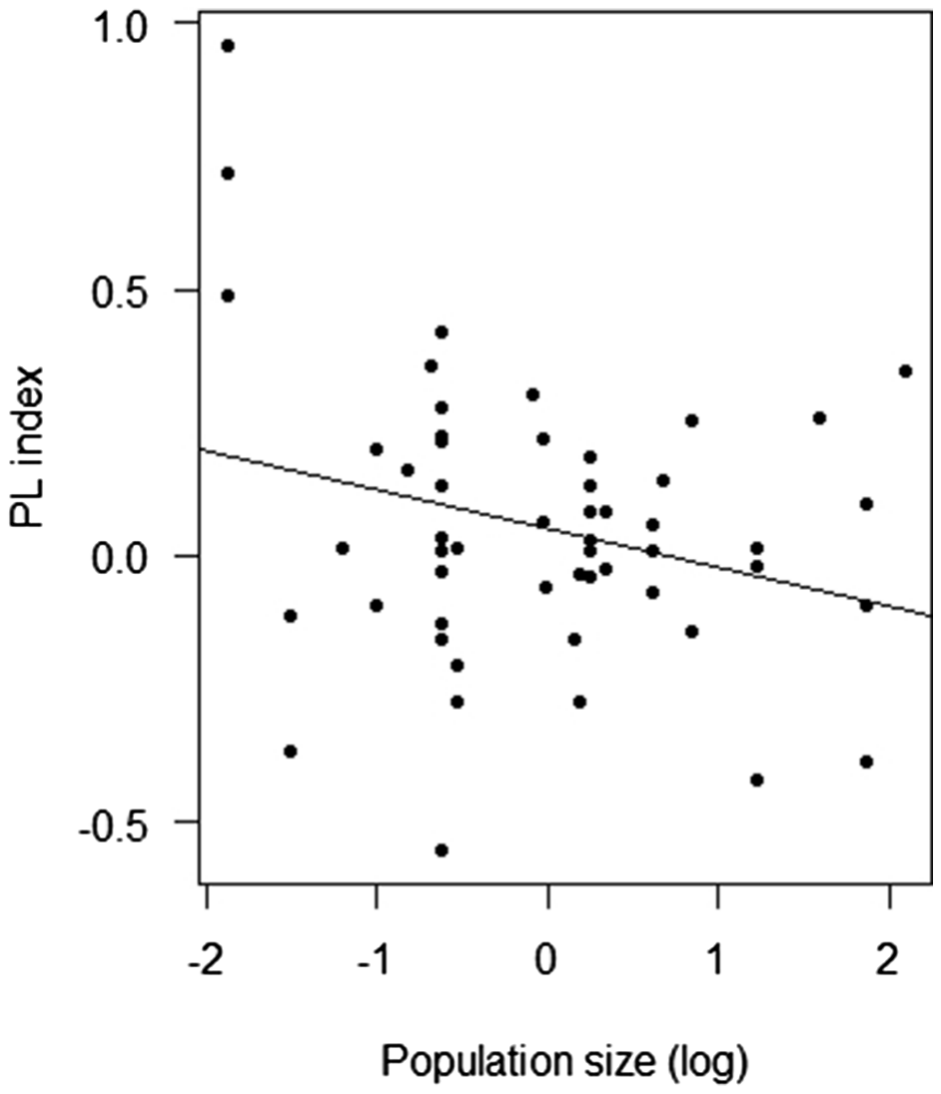
Results of linear regression analysis testing the response of the pollen limitation (PL) index of *Polemonium caeruleum* in each population in each year to population size.

**Figure 6:**
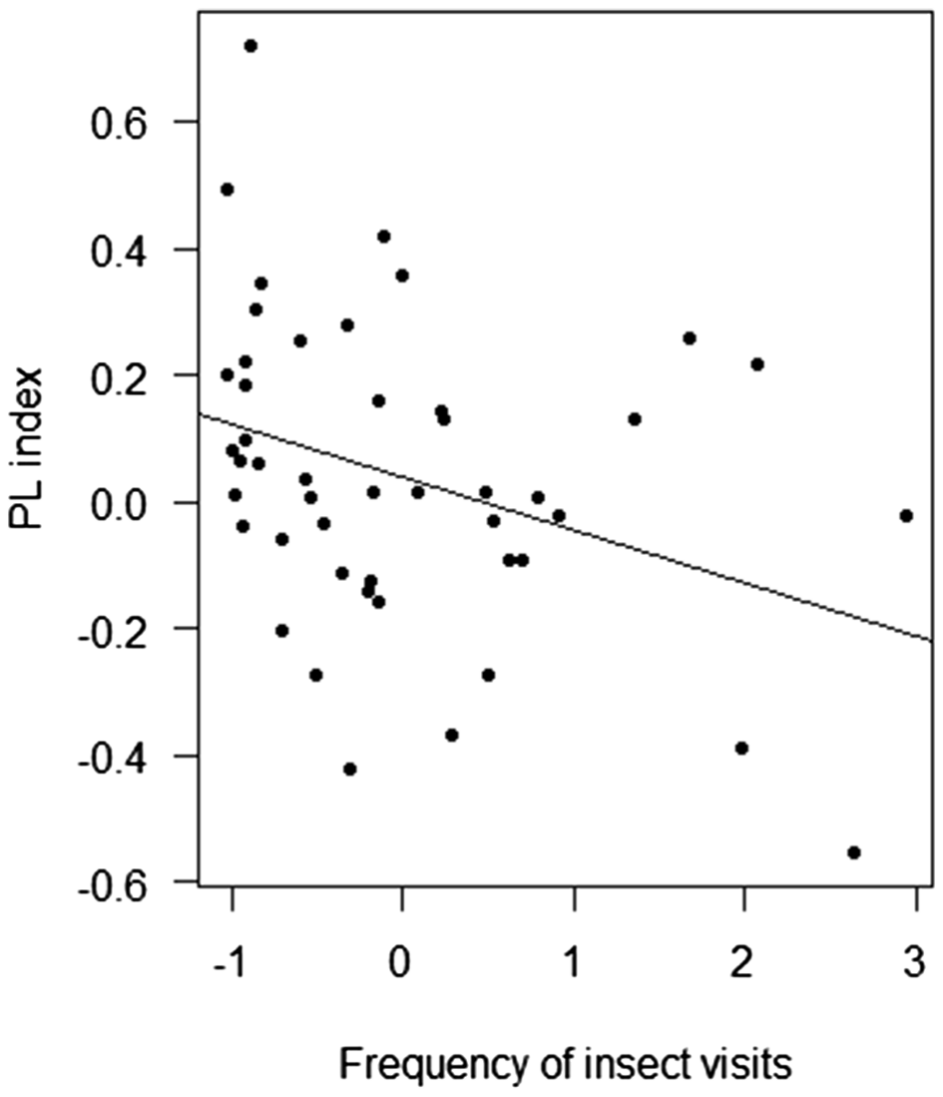
Results of linear regression analysis testing the response of the pollen limitation (PL) index of *Polemonium caeruleum* in each population in average frequency of insect visits.

Overall, the PL index was negatively affected by population size (F-statistic = 4.54, DF = 53, *p* = 0.038; Figure 5) and the frequency of insect visitation (F-statistic = 5.93, DF = 45, *p* = 0.019; Figure 6). However, the frequency of insect visitation was not associated with population size (F-statistic = 0.002, DF = 47, *p* = 0.962).

## Discussion

### Pollen Limitation

The present study demonstrated that *P. caeruleum* is pollen limited, but only to a small extent, at least in the Polish range of its distribution. Moreover, we observed spatiotemporal variations in pollen limitation, but with no obvious trends. These results are consistent with previous reports on pollen limitation in the Polish populations of this species, indicating that some populations suffer from pollen limitation; however, this phenomenon depends on population and year (Zych et al., 2013; Ostrowiecka et al., 2017). In the present study, the PL index markedly varied among populations and years, ranging from −0.77 to 0.96, and over 40% of the values were below zero. Surprisingly, in some populations, hand-pollinated flowers produced fewer seeds than naturally pollinated flowers. According to Young and Young (1992), this result can be explained by several facts, such as stigma damage during hand pollination, negative effects of high pollen density on the stigma on pollen tube growth in the style (stigma clogging), lower diversity of pollen donors in hand-pollinated flowers, and pollen removal by insects from the stigma of hand-pollinated flowers. Although we cannot rule out any of the above possibilities, our observations of insect activity suggest that insects visiting flowers treat the stigmas covered with pollen as a food source.

In three of the analysed populations (in one of them during 2 years), control flowers produced significantly fewer seeds than hand-pollinated flowers. Among these populations, two were small (BBR and SPN, with 10 and 90 flowering shoots, respectively, in the corresponding year) and one was large (MAL, with 6,000 flowering shoots in the corresponding year). The presence of pollen limitation in the large population may be the result of a sudden increase in the number of flowering individuals (probably as an effect of mowing) and consequent increase in the visitation frequency of insects, particularly honeybees, collecting large amounts of pollen, which may not left enough to pollination of other flowers (for details see next subsection “Flower visitors and their effects on seed set and pollen limitation”).

In contrast, in three other populations, control flowers produced significantly more seeds than hand-pollinated flowers. Among these populations, two were small (SIE and BIA, with 20 and 100 flowering shoots, respectively, in the corresponding year) and one was medium (ROS, with 120 flowering shoots in the corresponding year). In these three populations, insects likely treated the profusely hand-pollinated stigma as a food source. Additionally, apiaries are present close to the two small populations; and in BIA alone, honeybees were almost exclusive flower visitors and the frequency of their visitation was also high. The medium population was surrounded by a fen, with few other co-flowering plants; thus, stigmas covered with pollen may have been an attractive food source.

The magnitude of pollen limitation depends, among other factors, on the plant mating system; as such, self-compatible plants and generalists are less prone to pollen limitation than self-incompatible plants and specialists (Ashman et al., 2004; Knight et al., 2005; Wolowski et al., 2014). The generalist pollination system of *P. caeruleum* may reduce the probability of pollen limitation in populations, as has been shown in other plant species (Knight et al., 2005; Gómez et al., 2010; Wolowski et al., 2014). The *P. caeruleum* populations analysed in the present study are overall characterised by the presence of mixed mating systems (unpublished data), which rather does not predispose them to a high degree of pollen limitation.

### Flower Visitors and Their Effects on Seed Set and Pollen Limitation

The pollination system of *P. caeruleum* is described as generalist. In this plant, the cup-shaped flowers, which offer nectar and pollen, are easily accessible to a wide spectrum of insects. In the present study, we recorded visits by diverse groups of pollinators. In 3 of the 15 study populations, the assemblages of insects visiting flowers have been previously analysed. According to Zych et al. (2013), bumblebees were the most frequent and efficient visitors in KLE. Meanwhile, Ostrowiecka et al. (2017) observed that while KOP was visited by a diverse assemblage of insects, ZED was mainly visited by honeybees. In the present study, we observed a similar assemblage composition of insect visitors in these three populations in all seasons examined. However, this was not true for the rest of the studied populations. As such, the composition of insect assemblages varied across years, indicating spatiotemporal variations in the potential pollinator assemblages of *P. caeruleum*.

Considering data across all seasons and populations, honeybees were the most frequent visitors of *P. caeruleum* flowers, and together with bumblebees, they accounted for nearly 88% of total insect visits. Other groups of insects, although present in most populations, accounted for over 12% of all visits to flowers. Among these, flies (including hoverflies) were the largest contributors, whereas solitary bees, beetles, butterflies, and other insects contributed to a lesser extent. These trends support the previous assumption of Zych et al. (2013) that in terms of the pollination system, regardless of the high apparent generalisation, the realised generalisation is rather low among the *P. caeruleum* populations studied. Several groups of insects willingly used the food sources offered by *P. caeruleum* flowers, but only when honeybees and bumblebees were sparse.

The high proportion of honeybees (70.5%) among insects visiting *P. caeruleum* flowers is a consequence of the widespread introduction of colonies of these insects by beekeepers. Populations distant to settlements, such as those located in national parks (KOP, SPN, and WNP), were characterised by a lower frequency of honeybees visits and a more diverse composition of flower visitors assemblage. This result suggests the negative effect of honeybees on the diversity of other pollinators visiting flowers, corroborating previous reports (Aslan et al., 2016; Mallinger et al., 2017). Honeybees are highly competitive and may disrupt interactions of other native pollinators with plants (Goulson, 2003; Thomson, 2016; Valido et al., 2019; Milner et al., 2020; Angelella et al., 2021). The authors’ personal observations are that in populations characterised by a high visitation frequency of honeybees, pollen was removed from anthers very efficiently and rapidly, often resulting in difficulties in finding pollen for the hand-pollination of experimental flowers. A negligible amount of available pollen reduces the effectiveness of pollen transmission by insects and may thus reduce the seed set.

The GLMM showed that the visitation frequency of honeybees negatively affected seed production in open-pollinated flowers. However, despite the negative effect of honeybees on seed number in control treatment, among other insect groups visiting flowers, only honeybee visitation frequency showed a significant negative effect on the PL index, suggesting an important role of these insects as pollinators of *P. caeruleum*. This result is confirmed by previous findings that honeybees, in addition to bumblebees, are the key pollinator groups based on the pollen load carried by individuals and the frequency of their visitation (Zych et al., 2013). We assumed that the influence of honeybees on pollination of *P. caeruleum* may change with the number of insects visiting the population. In higher densities, those insects remove pollen from anthers very efficiently, and probably at some point, due to lack of available pollen to pollination, it may negatively influence the reproductive success of *P. caeruleum*. It is also possible that the observed negative impact of honeybees on seed set in control treatment is related to the collection of pollen grains from the stigmas by those insects, as evidenced by the fact that the PL index often took negative values. However, the negative PL index values may arise from the delivery of pollen of inadequate quality to the stigma (e.g., incompatible) of hand-pollinated flowers, or stigma damage during the hand-pollination by the experimenters. Consistently, honeybees appear to play a dual role as insects that on the one hand may in fact reduce pollen limitation, as reported previously (González-Varo et al., 2009; Tscheulin and Petanidou, 2011), while on the other hand may be a competitive species that collect and remove pollen from the population and in consequence preclude efficient pollination of plants.

Furthermore, hoverflies visits, although not recorded in all populations or years, positively affected seed set in control treatment. Hoverflies can have a strong positive effect on seed set (Vance et al., 2004), and in some plant species characterised by open flowers, these insects are more efficient pollinators than bumblebees (Fontaine et al., 2006). Moreover, hoverflies can carry pollen over longer distances than bees (Lysenkov, 2009), which may increase the probability of cross-pollination with the pollen of unrelated individuals, resulting in the production of more seeds of better quality, in *P. caeruleum*. Additionally, our observations indicate that flies, including hoverflies, visited fewer flowers within an inflorescence than bees, which may limit the deposition of pollen from the same individual.

Unlike the previous results of Zych et al. (2013) that reported bumblebees as the most efficient pollinators of *P. caeruleum*, this study did not reveal that higher bumblebee visitation rate influence the reproductive success of this species. However, Zych et al. (2013) studied only a single population, where these insects accounted for most of the visits. In contrast, our results showed that insect assemblages vary spatially and temporally and may be shaped by beekeeping activities, to some degree. Apart from human activity, including beekeeping, also other factors, such as the immediate surroundings, type of habitat and population size of *P. caeruleum*, as well as the presence and abundance of other groups of insects, and meteorological conditions in a given year, probably influenced the spatial and temporal diversity of insects visiting *P. caeruleum* flowers (Somme et al., 2014; Paajanen and Cronk, 2020; Rohde and Pilliod, 2021).

We observed that bees and, to a lesser degree, flies were the frequent visitors of *P. caeruleum* flowers. Likewise, a previous study (Zych et al., 2013) showed that these insect groups carried considerable *P. caeruleum* pollen loads. As the mean number of ovules in *P. caeruleum* flowers is rather low (*n* = 29), all morphogroups are expected to serve as efficient pollinators.

In *P. caeruleum*, the lack of specialisation in terms of pollination biology and the spatiotemporal variations in the composition of pollinator assemblages may result in high divergence in flower traits related to pollination. Our previous study on the chemistry of *P. caeruleum* nectar showed high variability in these traits among populations (Ryniewicz et al., 2020). We also observed some divergence in terms of the anatomy of *P. caeruleum* flowers, such as differences in flower size or the arrangement of reproductive parts. We assume that the morphology of *P. caeruleum* flowers shows certain adaptations to specific groups of pollinators; however, because of the high instability of the environment, these adaptative traits are not fixed, and such a variability of floral traits can be interpreted as “adaptive wandering” (Zych et al., 2019). In addition, the selection of randomly appearing features in *P. caeruleum* populations may be disrupted due to the presence of honeybees, which depend on human activity; this may affect the presence and diversity of other pollinators. Unpredictable visitation of flowers is a common feature among generalist plant species (Ghazoul, 2005; Ollerton et al., 2007; Wolowski et al., 2014), and this may lead to the development of self-compatibility (Arista et al., 2017). Local adaptations and variable degree of self-compatibility in *P. caeruleum* populations warrant further research.

### Effects of Meteorological Conditions on Seed Set and Pollen Limitation

The present study revealed that meteorological factors significantly affect seed production in control treatment and the PL index. Seed set was negatively affected by average monthly temperature in September of the previous season, when plants start to enter a state of dormancy and developed cold hardiness. In addition, seed set was negatively affected by average monthly precipitation in August of the previous season but positively affected by average monthly precipitation in June of the same season, when the field experiments were conducted. In case of meteorological factors which were analysed for the impact on the PL index, we observed only the influence of average monthly temperature in October, which increased the PL index.

*P. caeruleum* is a boreal plant species that prefers moist soils and does not tolerate well high temperatures, and a seasonal drought, during the vegetative season. All these weather extremes are becoming increasingly frequent and common in Poland (Kundzewicz and Matczak, 2012), posing a severe threat to the development of *P. caeruleum* populations. Our results indicate that unfavourable meteorological conditions during late summer and autumn the strongest affected the reproductive success and pollen limitation in populations of *P. caeruleum* (in the next season). Higher temperatures during autumn delay the development of cold hardiness (McKenzie and McLean, 1980) and can render plants weak, predisposing them to damage during winter.

This result supports the notion that temperature is an important factor shaping *P. caeruleum* distribution. On the other hand, the positive influence of average monthly precipitation in June, during *P. caeruleum* flowering and seed set indicate that the accessibility of soil water is especially important in this crucial time for plant reproduction.

However, there is some uncertainty in the context of the effect of monthly meteorological conditions. For instance, we observed differences in phenology among populations and years. Occasionally, the distance of some populations from the nearest weather station was over 40 km. Moreover, in addition to reproduction, favourable external conditions are essential for other activities, such as vegetative growth or processes related to extending survival (Sletvold and Gren, 2011; Sletvold and Ågren, 2015).

### Effect of Population Size on Seed Production and Pollen Limitation

Finally, population size affected some of the measured parameters. *P. caeruleum* populations were selected at the beginning of the project considering their sizes: five small, five medium, and five large; however, over the course of the study, the number of individuals in most populations changed significantly (Supplementary Table S1).

The effects of plant population size on seed production and pollen limitation through pollinator activity are well-known. For instance, this may be attributed to the lower attractiveness of small populations characterised by lower resources and/or deposition of higher amounts of heterospecific pollen on the stigma, particularly among generalist plant species (Jennersten and Nilsson, 1993; Ågren, 1996; Ashman, 2005; Knight et al., 2005; Aizen and Harder, 2007; Morales and Traveset, 2008). Although our results did not confirm the effect of population size on the visitation frequency of all insects, its effects on the seed set in control treatment and PL index were evident. Moreover, the visitation frequency of all insects negatively affected the PL index. Of note, honeybees and bumblebees, which were responsible for nearly 90% of all visits to *P. caeruleum* flowers, are characterised by flower constancy. The decrease in plant population and availability of limited food resources may be perceived by these insects as a severe threat, forcing the major pollinators to switch to different food sources. This may further worsen pollen limitation, ultimately leading to the complete disappearance of plant population.

Despite the wide array of factors tested in our study, still many of them, which are potentially important in determining degree of pollen limitation were not analysed in the present study, including uneven distribution of resources in individual parts of the inflorescence (Strelin and Aizen, 2018), species richness of co-flowering plants, interspecific competition to attract pollinators (Bell et al., 2005; Vamosi et al., 2006), resource availability (Haig and Westoby, 1988; Ashman et al., 2004), and habitat traits (Somme et al., 2014). Moreover, reproduction costs may differ between populations (Sletvold and Gren, 2011), which is also likely to be the case for *P. caeruleum*, as evidenced by the effect of populations on seed production as per our GLMM.

Long-term studies on pollen limitation are paramount for identifying trends in population dynamics and establishing the direction of change. Our results underscore the risk of further decline in small populations of *P. caeruleum*, which are subjected to more severe pollen limitation. This may decrease the genetic diversity in populations, ultimately leading to their complete disappearance. The present study may serve as the scientific basis for the conservation of *P. caeruleum* in its Polish range.

## Conclusion

The present study demonstrated that pollen limitation, seed production, insect visitation frequency, and insect assemblage composition exhibit spatiotemporal variations among *P. caeruleum* populations. Pollen limitation was rarely detected in *P. caeruleum* populations in the Polish range, and when present, it was enhanced by the small size of the population and the low frequency of pollinator visitation. Our results also imply a dual role of honeybees: while these insects are the most frequent flower visitors that decrease the PL index, their activity also reduces seed set of open-pollinated flowers. It can be a consequence of efficient pollen removal from *P. caeruleum*’s populations by honeybees, especially when flower visitation rate by those insects is very high.

As a generalist plant, *P. caeruleum* can be pollinated by diverse insect groups; however, decrease in the diversity of insects visiting flowers may affect the stability of plant populations. Moreover, decline in the number of plants in a population may reduce its attractiveness to honeybees and bumblebees, which are the major pollinators, eventually leading to the disappearance of the plant population.

Long-term study focusing on the relationship between plants and their pollinators allows us to better understand the processes taking place in plant populations (which is especially important in case of shrinking ones) and may help in more effective protection of endangered plant species.

## Data Availability Statement

The raw data supporting the conclusions of this article will be made available by the authors, without undue reservation.

## Author Contributions

JR and MZ conceived the study. JR, KR, MZ, EB, AW, BO, PM, IT, EJ, and MS assembled field data. JR and PM analysed the data. JR and MS visualised the data. MZ acquired funding. JR wrote the draft version of paper. All authors contributed to the article and approved the submitted version.

## Funding

The study was financially supported by the Polish National Science Centre (grant no. 2014/15/B/NZ8/00249 to MZ) and Faculty of Biology of the University of Warsaw (grant DSM no. 110134 to KR).

## Conflict of Interest

The authors declare that the research was conducted in the absence of any commercial or financial relationships that could be construed as a potential conflict of interest.

## Publisher’s Note

All claims expressed in this article are solely those of the authors and do not necessarily represent those of their affiliated organizations, or those of the publisher, the editors and the reviewers. Any product that may be evaluated in this article, or claim that may be made by its manufacturer, is not guaranteed or endorsed by the publisher.

## Footnotes

^1^ https://www.imgw.pl

## References

[ref1] Ågren J. (1996). Population size, pollinator limitation, and seed set in the self-incompatible herb *Lythrum salicaria*. Ecology 77, 1779–1790. doi: 10.2307/2265783

[ref2] Aizen M. A. Harder L. D. (2007). Expanding the limits of the pollen-limitation concept: effects of pollen quantity and quality. Ecology 88, 271–281. doi: 10.1890/06-1017, PMID: 17479745

[ref3] Angelella G. M. McCullough C. T. O’Rourke M. E. (2021). Honey bee hives decrease wild bee abundance, species richness, and fruit count on farms regardless of wildflower strips. Sci. Rep. 11:3202. doi: 10.1038/s41598-021-81967-1, PMID: 33547371 PMC7865060

[ref4] Arista M. Berjano R. Viruel J. Ortiz M. A. Talavera M. Ortiz P. L. (2017). Uncertain pollination environment promotes the evolution of a stable mixed reproductive system in the self-incompatible *Hypochaeris salzmanniana* (Asteraceae). Ann. Bot. 120, 447–456. doi: 10.1093/aob/mcx059, PMID: 28911017 PMC5591423

[ref5] Ashman T. L. (2005). The limits on sexual dimorphism in vegetative traits in a gynodioecious plant. Am. Nat. 166, S5–S16. doi: 10.1086/444598, PMID: 16224712

[ref6] Ashman T. L. Knight T. M. Steets J. A. Amarasekare P. Burd M. Campbell D. R.. (2004). Pollen limitation of plant reproduction: ecological and evolutionary causes and consequences. Ecology 85, 2408–2421. doi: 10.1890/03-8024

[ref7] Aslan C. E. Liang C. T. Galindo B. Hill K. Topete W. (2016). The role of honey bees as pollinators in natural areas. Nat. Areas J. 36, 478–488. doi: 10.3375/043.036.0413

[ref8] Baskin J. M. Baskin C. C. (2018). Pollen limitation and its effect on seed germination. Seed Sci. Res. 28, 1–8. doi: 10.1017/S0960258518000272

[ref9] Bell J. M. Karron J. D. Mitchell R. J. (2005). Interspecific competition for pollination lowers seed production and outcrossing in *Mimulus ringens*. Ecology 86, 762–771. doi: 10.1890/04-0694

[ref10] Borghi M. Perez de Souza L. Yoshida T. Fernie A. R. (2019). Flowers and climate change: a metabolic perspective. New Phytol. 224, 1425–1441. doi: 10.1111/nph.16031, PMID: 31257600

[ref11] Brooks M. E. Kristensen K. van Benthem K. J. Magnusson A. Berg C. W. Nielsen A.. (2017). glmmTMB balances speed and flexibility among packages for zero-inflated generalized linear mixed modeling. R J. 9, 378–400. doi: 10.32614/rj-2017-066

[ref12] Fernández J. D. Bosch J. Nieto-Ariza B. Gómez J. M. (2012). Pollen limitation in a narrow endemic plant: geographical variation and driving factors. Oecologia 170, 421–431. doi: 10.1007/s00442-012-2312-1, PMID: 22492167

[ref13] Fontaine C. Dajoz I. Meriguet J. Loreau M. (2006). Functional diversity of plant-pollinator interaction webs enhances the persistence of plant communities. PLoS Biol. 4:e1. doi: 10.1371/journal.pbio.0040001, PMID: 16332160 PMC1310649

[ref14] Forrest J. R. K. (2015). Plant-pollinator interactions and phenological change: what can we learn about climate impacts from experiments and observations? Oikos 124, 4–13. doi: 10.1111/oik.01386

[ref15] Gallagher M. K. Campbell D. R. (2017). Shifts in water availability mediate plant–pollinator interactions. New Phytol. 215, 792–802. doi: 10.1111/nph.14602, PMID: 28517023

[ref16] Ghazoul J. (2005). Pollen and seed dispersal among dispersed plants. Biol. Rev. Camb. Philos. Soc. 80:413. doi: 10.1017/S1464793105006731 16094807

[ref17] Gómez J. M. Abdelaziz M. Lorite J. Muñoz-Pajares A. J. Perfectti F. (2010). Changes in pollinator fauna cause spatial variation in pollen limitation. J. Ecol. 98, 1243–1252. doi: 10.1111/j.1365-2745.2010.01691.x

[ref18] Gómez J. M. Bosch J. Perfectti F. Fernández J. Abdelaziz M. (2007). Pollinator diversity affects plant reproduction and recruitment: The tradeoffs of generalization. Oecologia 153, 597–605. doi: 10.1007/s00442-007-0758-3, PMID: 17576602

[ref19] González-Varo J. P. Arroyo J. Aparicio A. (2009). Effects of fragmentation on pollinator assemblage, pollen limitation and seed production of Mediterranean myrtle (*Myrtus communis*). Biol. Conserv. 142, 1058–1065. doi: 10.1016/j.biocon.2009.01.017

[ref20] Goulson D. (2003). Effects of introduced bees on native ecosystems. Annu. Rev. Ecol. Evol. Syst. 34, 1–26. doi: 10.1146/annurev.ecolsys.34.011802.132355

[ref21] Haig D. Westoby M. (1988). On limits to seed production. Am. Nat. 131, 757–759. doi: 10.1086/284817

[ref22] Hallmann C. A. Sorg M. Jongejans E. Siepel H. Hofland N. Schwan H.. (2017). More than 75 percent decline over 27 years in total flying insect biomass in protected areas. PLoS One 12:e0185809. doi: 10.1371/journal.pone.0185809, PMID: 29045418 PMC5646769

[ref23] Hosmer D. W. Lemeshow S. (2000). Applied Logistic Regression. John Wiley & Sons, Inc. New York.

[ref24] Jennersten O. Nilsson S. G. (1993). Insect flower visitation frequency and seed production in relation to patch size of *Viscaria vulgaris* (Caryophyllaceae). Oikos 68:283. doi: 10.2307/3544841

[ref25] Kaźmierczakowa R. Bloch-Orłowska J. Celka Z. Cwener A. Michalska-Hejduk D. Pawlikowski P.. (2016). Polska Czerwona Lista paprotników I roślin Kwiatowych (Polish Red List of Pteridophytes and Flowering Plants). Instytut Ochrony Przyrody Polskiej Akademii Nauk, Kraków.

[ref26] Knight T. M. Steets J. A. Vamosi J. C. Mazer S. J. Burd M. Campbell D. R.. (2005). Pollen limitation of plant reproduction: pattern and process. Annu. Rev. Ecol. Evol. Syst. 36, 467–497. doi: 10.1146/annurev.ecolsys.36.102403.115320

[ref27] Kudo G. Ida T. Y. (2013). Early onset of spring increases the phenological mismatch between plants and pollinators. Ecology 94, 2311–2320. doi: 10.1890/12-2003.1, PMID: 24358716

[ref28] Kundzewicz Z. W. Matczak P. (2012). Climate change regional review: Poland. Wiley Interdiscip. Rev. Clim. Chang. 3, 297–311. doi: 10.1002/wcc.175

[ref29] Lister B. C. Garcia A. (2018). Climate-driven declines in arthropod abundance restructure a rainforest food web. Proc. Natl. Acad. Sci. U. S. A. 115, E10397–E10406. doi: 10.1073/pnas.1722477115, PMID: 30322922 PMC6217376

[ref30] Liu Y. Mu J. Niklas K. J. Li G. Sun S. (2012). Global warming reduces plant reproductive output for temperate multi-inflorescence species on the Tibetan plateau. New Phytol. 195, 427–436. doi: 10.1111/j.1469-8137.2012.04178.x, PMID: 22591333

[ref31] Lysenkov S. N. (2009). On the estimation of the influence of the character of insect pollinators movements on the pollen transfer dynamics. Entomol. Rev. 89, 143–149. doi: 10.1134/S0013873809020031

[ref32] Mallinger R. E. Gaines-Day H. R. Gratton C. (2017). Do managed bees have negative effects on wild bees?: A systematic review of the literature. PLoS One 12:e0189268. doi: 10.1371/journal.pone.0189268, PMID: 29220412 PMC5722319

[ref33] McKenzie J. S. McLean G. E. (1980). Some factors associated with injury to alfaalfa during the 1977–78 winter at Beaverlodge, Alberta. Can. J. Plant Sci. 60, 103–112. doi: 10.4141/cjps80-015

[ref34] Milner J. R. D. Bloom E. H. Crowder D. W. Northfield T. D. (2020). Plant evolution can mediate negative effects from honey bees on wild pollinators. Ecol. Evol. 10, 4407–4418. doi: 10.1002/ece3.6207, PMID: 32489606 PMC7246215

[ref35] Morales C. L. Traveset A. (2008). Interspecific pollen transfer: Magnitude, prevalence and consequences for plant fitness. Crit. Rev. Plant Sci. 27, 221–238. doi: 10.1080/07352680802205631

[ref36] Motten A. F. (1986). Pollination ecology of the spring wildflower community of a temperate deciduous forest. Ecol. Monogr. 56, 21–42. doi: 10.2307/2937269

[ref37] Ollerton J. Killick A. Lamborn E. Watts S. Whiston M. (2007). Multiple meanings and modes: on the many ways to be a generalist flower. Taxon 56, 717–728. doi: 10.2307/25065855

[ref38] Ollerton J. Winfree R. Tarrant S. (2011). How many flowering plants are pollinated by animals? Oikos 120, 321–326. doi: 10.1111/j.1600-0706.2010.18644.x

[ref39] Ostrowiecka B. Brzosko E. Jermakowicz E. Wróblewska A. Mirski P. Roguz K.. (2017). Breeding system variability, pollination biology, and reproductive success of rare *Polemonium caeruleum* L. in NE Poland. Acta Agrobot. 70:1709. doi: 10.5586/aa.1709

[ref40] Paajanen M. P. T. Cronk Q. (2020). Moth versus fly: A preliminary study of the pollination mode of two species of endemic Asteraceae from St Helena (*Commidendrum robustum* and *C. rugosum*) and its conservation implications. Biodivers. Data J. 8:e52057. doi: 10.3897/BDJ.8.E52057, PMID: 32431561 PMC7220971

[ref41] Pigott C. D. (1958). *Polemonium caeruleum* L. J. Ecol. 46, 507–525. doi: 10.2307/2257416

[ref42] Rohde A. T. Pilliod D. S. (2021). Spatiotemporal dynamics of insect pollinator communities in sagebrush steppe associated with weather and vegetation. Glob. Ecol. Conserv. 29:e01691. doi: 10.1016/j.gecco.2021.e01691

[ref43] Rutkowski L. (2000). “*Polemonium caeruleum* L. Wielosił błękitny” in Polska czerwona księga roślin. Paprotniki i rośliny kwiatowe. eds. Kaźmierczakowa R. Zarzycki K. (Kraków: Instytut Botaniki im. W. Szafera PAN i Instytut Ochrony Przyrody PAN), 310–311.

[ref44] Ryniewicz J. Skłodowski M. Chmur M. Bajguz A. Roguz K. Roguz A.. (2020). Intraspecific variation in nectar chemistry and its implications for insect visitors: The case of the medicinal plant, polemonium caeruleum l. Plan. Theory 9:1297. doi: 10.3390/plants9101297, PMID: 33019586 PMC7600102

[ref45] Sletvold N. Ågren J. (2015). Nonlinear costs of reproduction in a long-lived plant. J. Ecol. 103, 1205–1213. doi: 10.1111/1365-2745.12430

[ref46] Sletvold N. Gren J. A. (2011). Nonadditive effects of floral display and spur length on reproductive success in a deceptive orchid. Ecology 92, 2167–2174. doi: 10.1890/11-0791.1, PMID: 22352154

[ref47] Somme L. Mayer C. Jacquemart A. L. (2014). Multilevel spatial structure impacts on the pollination services of *Comarum palustre* (Rosaceae). PLoS One 9:e99295. doi: 10.1371/journal.pone.0099295, PMID: 24915450 PMC4051681

[ref48] Stephens P. A. Sutherland W. J. (1999). Consequences of the Allee effect for behaviour, ecology and conservation. Trends Ecol. Evol. 14, 401–405. doi: 10.1016/S0169-5347(99)01684-5, PMID: 10481204

[ref49] Strelin M. M. Aizen M. A. (2018). The interplay between ovule number, pollination and resources as determinants of seed set in a modular plant. PeerJ 6:e5384. doi: 10.7717/peerj.5384, PMID: 30083472 PMC6074782

[ref50] Thomas C. D. Jones T. H. Hartley S. E. (2019). “Insectageddon”: A call for more robust data and rigorous analyses. Glob. Chang. Biol. 25, 1891–1892. doi: 10.1111/gcb.14608, PMID: 30821400

[ref51] Thomson D. M. (2016). Local bumble bee decline linked to recovery of honey bees, drought effects on floral resources. Ecol. Lett. 19, 1247–1255. doi: 10.1111/ele.12659, PMID: 27539950

[ref52] Tscheulin T. Petanidou T. (2011). Does spatial population structure affect seed set in pollen-limited *Thymus capitatus*? Apidologie 42, 67–77. doi: 10.1051/apido/2010035

[ref53] Valido A. Rodríguez-Rodríguez M. C. Jordano P. (2019). Honeybees disrupt the structure and functionality of plant-pollinator networks. Sci. Rep. 9:4711. doi: 10.1038/s41598-019-41271-5, PMID: 30886227 PMC6423295

[ref54] Vamosi J. C. Knight T. M. Steets J. A. Mazer S. J. Burd M. Ashman T. L. (2006). Pollination decays in biodiversity hotspots. Proc. Natl. Acad. Sci. U. S. A. 103, 956–961. doi: 10.1073/pnas.0507165103, PMID: 16418284 PMC1347978

[ref55] van der Sluijs J. P. (2020). Insect decline, an emerging global environmental risk. Curr. Opin. Environ. Sustain. 46, 39–42. doi: 10.1016/j.cosust.2020.08.012

[ref56] Vance N. C. Bernhardt P. Edens R. M. (2004). Pollination and seed production in *Xerophyllum tenax* (Melanthiaceae) in the Cascade Range of Central Oregon. Am. J. Bot. 91, 2060–2068. doi: 10.3732/ajb.91.12.2060, PMID: 21652355

[ref57] Wagner D. L. Grames E. M. Forister M. L. Berenbaum M. R. Stopak D. (2021). Insect decline in the Anthropocene: death by a thousand cuts. Proc. Natl. Acad. Sci. U. S. A. 118:e2023989118. doi: 10.1073/PNAS.2023989118, PMID: 33431573 PMC7812858

[ref58] Warren M. S. Maes D. van Swaay C. A. M. Goffart P. van Dyck H. Bourn N. A. D.. (2021). The decline of butterflies in Europe: problems, significance, and possible solutions. Proc. Natl. Acad. Sci. U. S. A. 118:e2002551117. doi: 10.1073/PNAS.2002551117, PMID: 33431566 PMC7812787

[ref59] Waser N. M. Chittka L. Price M. V. Williams N. M. Ollerton J. (1996). Generalization in pollination systems, and why it matters. Ecology 77, 1043–1060. doi: 10.2307/2265575

[ref60] Wolowski M. Ashman T. L. Freitas L. (2014). Meta-analysis of pollen limitation reveals the relevance of pollination generalization in the Atlantic forest of Brazil. PLoS One 9:e89498. doi: 10.1371/journal.pone.0089498, PMID: 24586827 PMC3931788

[ref61] Young L. W. Wilen R. W. Bonham-Smith P. C. (2004). High temperature stress of *Brassica napus* during flowering reduces micro- and megagametophyte fertility, induces fruit abortion, and disrupts seed production. J. Exp. Bot. 55, 485–495. doi: 10.1093/jxb/erh038, PMID: 14739270

[ref62] Young H. J. Young T. P. (1992). Alternative outcomes of natural and experimental high pollen loads. Ecology 73, 639–647. doi: 10.2307/1940770

[ref63] Zhang L. J. Lou A. R. (2015). Pollen limitation in invasive populations of *Solanum rostratum* and its relationship to population size. J. Plant Ecol. 8, 154–158. doi: 10.1093/jpe/rtv013

[ref64] Ziemiański M. A. Zych M. (2016). Pollination biology of the urban populations of an ancient forest, spring ephemeral plant. Acta Soc. Bot. Pol. 85:3489. doi: 10.5586/asbp.3489

[ref65] Zuur A. F. Ieno E. N. Elphick C. S. (2010). A protocol for data exploration to avoid common statistical problems. Methods Ecol. Evol. 1, 3–14. doi: 10.1111/j.2041-210x.2009.00001.x

[ref66] Zych M. Junker R. R. Nepi M. Stpiczyńska M. Stolarska B. Roguz K. (2019). Spatiotemporal variation in the pollination systems of a supergeneralist plant: is *Angelica sylvestris* (Apiaceae) locally adapted to its most effective pollinators? Ann. Bot. 123, 415–428. doi: 10.1093/aob/mcy140, PMID: 30059963 PMC6344219

[ref67] Zych M. Stpiczyńska M. Roguz K. (2013). Reproductive biology of the red list species *Polemonium caeruleum* (Polemoniaceae). Bot. J. Linn. Soc. 173, 92–107. doi: 10.1111/boj.12071

