# Supplementary material for: Spatiotemporal Variations in Seed Set and Pollen Limitation in Populations of the Rare Generalist Species Polemonium caeruleum in Poland
Source: Front Plant Sci. 2022 Jan 3;12:755830. doi: 10.3389/fpls.2021.755830 (PMC8761629; doi:10.3389/fpls.2021.755830)
Supplement: Supplementary file 1 [file Table_1.DOCX]

**Supplementary Data. Table S1**

Location and characteristics of the 15 *Polemonium caeruleum* populations studied.

| **Population code** | **Coordinates** | **No. of flowering shoots in a given year** | | **Size class** | **Habitat** |
| --- | --- | --- | --- | --- | --- |
| BBR | 51°33'59" N  20°26'34" E | 2014 | 10 | small | wet meadow |
|  |  | 2015 | 10 |  |  |
|  |  | 2016 | 10 |  |  |
|  |  | 2017 | 10 |  |  |
| BIA | 52°41'20" N  23°52'42" E | 2015 | 100 | small | multispecies meadow |
|  |  | 2016 | 100 |  |  |
|  |  | 2017 | 100 |  |  |
|  |  | 2018 | 100 |  |  |
| BOB | 53°57'46" N  16°34'24" E | 2014 | 50 | medium | wet meadow |
|  |  | 2015 | 50 |  |  |
|  |  | 2016 | 310 |  |  |
|  |  | 2017 | 100 |  |  |
| CZL | 50°35'45" N  19°51'46" E | 2014 | 500 | medium | wet meadow surrounded by deciduous forest |
|  |  | 2015 | 500 |  |  |
|  |  | 2016 | 600 |  |  |
|  |  | 2017 | 500 |  |  |
| DRO | 52°33'07" N  22°27'36" E | 2015 | 100 | medium | meadow colonizing by trees and shrubs |
|  |  | 2016 | 270 |  |  |
|  |  | 2017 | 600 |  |  |
| KCZ | 54°22'44" N  18°19'13" E | 2014 | 300 | large | wet meadow |
|  |  | 2015 | 300 |  |  |
|  |  | 2016 | 1500 |  |  |
|  |  | 2017 | 1000 |  |  |
| KLE | 53°02'55" N  21°51'41" E | 2014 | 1000 | large | meadow |
|  |  | 2015 | 1000 |  |  |
|  |  | 2016 | 1500 |  |  |
|  |  | 2017 | 1100 |  |  |
| KOP | 53°15'55" N  22°36'38" E | 2015 | 100 | small | deciduous forest |
|  |  | 2016 | 100 |  |  |
|  |  | 2017 | 100 |  |  |
|  |  | 2018 | 100 |  |  |
| MAL | 50°49'36" N  20°18'19" E | 2014 | 500 | large | wet meadow |
|  |  | 2015 | 500 |  |  |
|  |  | 2016 | 420 |  |  |
|  |  | 2017 | 6000 |  |  |
| ORZ | 52°40'47" N  23°31'47" E | 2015 | 10000 | large | wet meadow |
|  |  | 2016 | 10000 |  |  |
|  |  | 2017 | 10000 |  |  |
|  |  | 2018 | 10000 |  |  |
| ROS | 53°54'40" N  22°56'17" E | 2015 | 120 | medium | fen |
|  |  | 2016 | 120 |  |  |
|  |  | 2017 | 120 |  |  |
|  |  | 2018 | 120 |  |  |
| SIE | 52°53'51" N  23°53'34" E | 2015 | 20 | small | a drainage ditch on the border of a meadow and a forest |
|  |  | 2016 | 20 |  |  |
|  |  | 2018 | 35 |  |  |
| SPN | 54°42'42" N  17°26'32" E | 2016 | 100 | small | sedgeland |
|  |  | 2017 | 90 |  |  |
|  |  | 2018 | 70 |  |  |
| WPN | 54°07'06" N  23°04'28" E | 2014 | 500 | medium | wet meadow surrounded by alder car and spruce forest |
|  |  | 2015 | 500 |  |  |
|  |  | 2016 | 450 |  |  |
|  |  | 2017 | 450 |  |  |
| ZED | 53°06'36" N  23°27'38" E | 2015 | 3000 | large | wet meadow |
|  |  | 2016 | 3000 |  |  |
|  |  | 2017 | 3000 |  |  |
|  |  | 2018 | 15000 |  |  |
