# Supplementary material for: Spatiotemporal Variations in Seed Set and Pollen Limitation in Populations of the Rare Generalist Species Polemonium caeruleum in Poland
Source: Front Plant Sci. 2022 Jan 3;12:755830. doi: 10.3389/fpls.2021.755830 (PMC8761629; doi:10.3389/fpls.2021.755830)
Supplement: Supplementary file 2 [file Table_2.DOCX]

**Supplementary Data. Table S2**

Zero-inflation generalized linear mixed models with negative binomial distribution and random effect of population testing response of seed set of *Polemonium caeruleum* to population size, average visitation rate of different insect groups, and meteorological conditions.

| Model | Parameters (+ random effect of Population) | AIC |
| --- | --- | --- |
| M1 | Population size, Bumblebee, Honeybee, Solitary bee, Hoverflies, Other flies, Butterflies, Other insects, Temperature in May, Precipitation in May, Temperature in June, Precipitation in June, Temperature in July, Precipitation in July, Temperature in of the August previous year, Precipitation in of the August previous year, Temperature in September of the previous year, Precipitation in September of the previous year, Temperature in October of the previous year Precipitation in October of the previous year | 4151.6 |
| M2 | Population size, Bumblebee, Honeybee, Solitary bee, Hoverflies, Other flies, Butterflies, Other insects, Precipitation in May, Temperature in June, Precipitation in June, Temperature in July, Precipitation in July, Temperature in of the August previous year, Precipitation in of the August previous year, Temperature in September of the previous year, Precipitation in September of the previous year, Temperature in October of the previous year Precipitation in October of the previous year | 4150.8 |
| M3 | Population size, Bumblebee, Honeybee, Solitary bee, Hoverflies, Other flies, Butterflies, Other insects, Precipitation in May, Temperature in June, Precipitation in June, Temperature in July, Precipitation in July, Precipitation in of the August previous year, Temperature in September of the previous year, Precipitation in September of the previous year, Temperature in October of the previous year Precipitation in October of the previous year | 4149.9 |
| M4 | Population size, Bumblebee, Honeybee, Solitary bee, Hoverflies, Other flies, Butterflies, Other insects, Precipitation in May, Precipitation in June, Temperature in July, Precipitation in July, Precipitation in of the August previous year, Temperature in September of the previous year, Precipitation in September of the previous year, Temperature in October of the previous year Precipitation in October of the previous year | 4148.1 |
