# Supplementary material for: Spatiotemporal Variations in Seed Set and Pollen Limitation in Populations of the Rare Generalist Species Polemonium caeruleum in Poland
Source: Front Plant Sci. 2022 Jan 3;12:755830. doi: 10.3389/fpls.2021.755830 (PMC8761629; doi:10.3389/fpls.2021.755830)
Supplement: Supplementary file 3 [file Table_3.DOCX]

**Supplementary Data. Table S3**

List of candidate models* that were taken into consideration in the model averaging procedure, for the assessment of influence of average visitation rate of different insect groups on the level of pollen limitation index.

| **Model** | **Parameters** | **ΔAICc** |
| --- | --- | --- |
| M0 | Population, Year | 0.00 |
| M1 | Population, Year, Bumblebee | 6.73 |
| M2 | Population, Year, Honeybee | 0.81 |
| M3 | Population, Year, Solitary bees | 7.06 |
| M4 | Population, Year, Hoverflies | 7.38 |
| M5 | Population, Year, Other flies | 5.91 |
| M6 | Population, Year, Butterflies | 6.83 |
| M7 | Population, Year, Beetles | 3.36 |
| M8 | Population, Year, Other insects | 3.72 |

* please note that including more than one insect group will result in the value of ΔAICc > 2
