# Supplementary material for: Spatiotemporal Variations in Seed Set and Pollen Limitation in Populations of the Rare Generalist Species Polemonium caeruleum in Poland
Source: Front Plant Sci. 2022 Jan 3;12:755830. doi: 10.3389/fpls.2021.755830 (PMC8761629; doi:10.3389/fpls.2021.755830)
Supplement: Supplementary file 4 [file Table_4.DOCX]

**Supplementary Data. Table S4**

List of candidate models* that were taken into consideration in the model averaging procedure, for the assessment of weather conditions influence on the level of pollen limitation index.

| **Model** | **Parameters** | **ΔAICc** |
| --- | --- | --- |
| M0 | Population, Year | 0.00 |
| M1 | Population, Year, Precipitation in May | 7.08 |
| M2 | Population, Year, Temperature in May | 6.18 |
| M3 | Population, Year, Precipitation in June | 7.14 |
| M4 | Population, Year, Temperature in June | 7.02 |
| M5 | Population, Year, Precipitation in July | 7.26 |
| M6 | Population, Year, Temperature in July | 6.16 |
| M7 | Population, Year, Precipitation in August of the previous year | 4.90 |
| M8 | Population, Year, Temperature in August of the previous year | 6.99 |
| M9 | Population, Year, Precipitation in September of the previous year | 6.96 |
| M10 | Population, Year Temperature in September of the previous year | 6.53 |
| M11 | Population, Year, Precipitation in October of the previous year | 5.63 |
| M12 | Population, Year, Temperature in October of the previous year | 0.85 |

* please note that including more than one insect group will result in the value of ΔAICc > 2
